# Application of PCR and Microscopy to Detect *Helicobacter pylori* in Gastric Biopsy Specimen among Acid Peptic Disorders at Tertiary Care Centre in Eastern Nepal

**DOI:** 10.1155/2019/3695307

**Published:** 2019-02-05

**Authors:** Nayanum Pokhrel, Basudha Khanal, Keshav Rai, Manish Subedi, Narayan Raj Bhattarai

**Affiliations:** ^1^Department of Microbiology, B. P. Koirala Institute of Health Sciences, Dharan, Nepal; ^2^Department of Internal Medicine, B. P. Koirala Institute of Health Sciences, Dharan, Nepal

## Abstract

**Background:**

* Helicobacter pylori* infection is most prevalent in developing countries. It is an etiological agent of peptic ulcer, gastric adenocarcinoma, and mucosal-associated lymphoid tissue (MALT) lymphoma. Despite the development of different assays to confirm *H. pylori* infection, the diagnosis of infection is challenged by precision of the applied assay. Hence, the aim of this study was to understand the diagnostic accuracy of PCR and microscopy to detect the *H. pylori* in the gastric antrum biopsy specimen from gastric disorder patients.

**Methods:**

A total of 52 patients with gastric disorders underwent upper gastrointestinal endoscopy with biopsy. The *H. pylori* infection in gastric biopsies was identified after examination by microscopy and 23S rRNA specific PCR. The agreement between two test results were analysed by McNemar's test and Kappa coefficient.

**Result:**

*H. pylori* infection was confirmed in 9 (17.30%) patients by both assays, 6.25% in antral gastritis, 22.22% in gastric ulcer, 100% in gastric ulcer with duodenitis, 50% in gastric ulcer with duodenal ulcer, and 33.33% in severe erosive duodenitis with antral gastritis. Out of nine *H. pylori* infection confirmed patients, 3 patients were confirmed by microscopy and 8 patients by PCR. In case of two patients, both microscopy and PCR assay confirmed the *H. pylori* infection. The agreement between two test results was 86.54% and disagreed by 13.46% (*p* value > 0.05).

**Conclusion:**

We found that PCR assay to detect *H. pylori* is more sensitive than microscopy. However, we advocate for the combination of both assays to increase the strength of diagnostic accuracy due to the absence of the gold standard assay for *H. pylori* infection.

## 1. Introduction

*Helicobacter pylori* (*H. pylori*) is a Gram-negative bacterium that plays a remarkable role in the causation of gastrointestinal diseases such as peptic ulcers, low-grade B-cell lymphoma (MALT lymphoma), and gastric cancer [1, 2]. Several epidemiological studies also evidenced that *H. pylori-*infected individuals showed the incidence of gastric carcinoma [3]. The discrepancy of *H. pylori* prevalence has been shown among different population as well as in different countries. In fact, the transmission of the infection is influenced by the socioeconomic conditions. About 90% prevalence have been reported in developing nations in comparison with 50% occurrence in developed countries [4, 5]. Moreover, both gastric cancer and peptic ulcer cause more than a million deaths per year globally, thus making it an important health issue [6, 7].

Diagnostic tests for *H. pylori* include invasive and noninvasive methods with the involved techniques being either direct or indirect. Microscopy detection of the bacteria and culture is a direct method whereas demonstration of urease production and detection of stool antigen or an antibody is considered an indirect method, which is used as a response marker of infectious diseases. Advancement in molecular methods is now used as a reliable tool for diagnosis of infectious diseases due to its increasing sensitivity and specificity [8]. Due to resource constraints, diagnosis by noninvasive tests such as urea breath test or invasive approach by bacterial culture of the biopsied tissue is not performed in our setting. Likewise, the reliability of immunological tests is always a matter of debate. In recent years, application of molecular method such as polymerase chain reaction (PCR) has revolutionized the diagnostic approaches for the detection of *H. pylori*. In addition, it also tracks the several genetic alteration in bacilli for understanding the drugs resistance characteristics [9] and coinfection of pathogens in gastric disease [10]. The molecular approach has also helped in comparative analysis between conventional methods such as microscopy and rapid urease test with PCR in resource-limited settings for effective diagnosis and treatment. In our setup with the advantage of the availability of molecular methods, we compared microscopy with PCR to see the effectiveness of each method for further evaluation of the study.

It is utmost important to identify *H. pylori* infection in gastroduodenal diseases so that the probable gastrointestinal malignancy can be prevented on time. In developing countries such as Nepal, the prevalence of *H. pylori* is notably higher in number of duodenal ulcer, gastric ulcer, and gastritis but a few data on burden of infections are available [11]. Therefore, this study has the aim to detect *H. pylori* in upper gastrointestinal endoscopic biopsy specimens by different diagnostic tools and evaluate the accuracy of *H. pylori* detecting tools in acid peptic disorder patients attending B. P. Koirala Institute of Health Sciences, Dharan.

## 2. Materials and Methods

### 2.1. Patients and Samples

This study was performed at B. P. Koirala Institute of Health Sciences (BPKIHS), Dharan, Nepal, from January 2017 to December 2017. Ethical clearance was obtained from the Institutional Review Committee (IRC-321/073/074) at BPKIHS. A written consent from 52 patients with symptoms of dyspepsia was taken before the biopsy specimen was collected for the study. The patient with age less than 14 years was excluded in this study. Likewise, the patients with history of long-term drugs known to cause gastritis such as steroids, anticoagulants, and lesions suggestive of malignancy on endoscopy were excluded from the study.

About 4 mm biopsy specimen from either the infected site or normal mucosa of the gastric antrum was collected. The tissue biopsy was cut with a sterile scalpel blade in a sterile Petri dish into two pieces. First specimen was preserved in normal saline and kept in a freezer at −80°C for PCR. Second tissue biopsy was processed for microscopic assessment [12]. In this study, storage of the biopsy specimens was done at −80°C which prevents the deterioration of DNA before the PCR analysis. In order to confirm the PCR inhibition, PCR-negative samples were diluted in 1 : 10 PCR grade water and PCR was repeated.

### 2.2. Microscopy

A smear was prepared by picking the biopsy specimen with a sterile swab and smeared onto two clean microscopic glass slides. After air-drying, the smear was fixed with uppermost flame of the Bunsen burner and allowed to cool. The smear was stained with the modified Gram-staining technique using carbol fuchsin as the counterstain. In the second glass slide, smear was fixed with methanol and Giemsa staining was performed [12].

### 2.3. PCR

The biopsy sample was taken out from the freezer and thawed at 37°C prior to processing the sample. The DNA extraction from the biopsy specimen was performed by using the Wizard Genomic DNA purification kit (Promega, Cat no. A1125) [13]. In brief, the biopsy was homogenized by a glass rod in nucleic lysis solution, and lysate was incubated at 65°C for 30 minutes and at 80°C for 5 minutes. After removal of protein precipitates from the lysate, the supernatant containing DNA was further precipitated in isopropanol and 70% ethanol, separately. Ethanol was gently removed, and pellet was air-dried. Finally, 100 *µ*L of DNA rehydration solution was added to rehydrate the DNA by incubating overnight at 4°C and stored at −20°C.

The *H. pylori-*specific PCR was performed to detect 23S rRNA gene [14]. PCR master mix was prepared in 25 *μ*l final volume which constituted 2 mM of MgCl_2_, 0.1 mg/ml of BSA, and 0.175 *µ*M of primer HP-23S-F (5′-AGATGGGAGCTGTCTCAACCAG-3′); 0.25 *µ*M of primer HP-23S-R (5′-TCCTGCGCATGATATTCCC-3′); and 0.2 mM DNTP mix, 0.5 unit of Hotstar Taq polymerase (Qiagen, Cat. nr. 203605), and 2.5 *µ*L of DNA template. PCR water was used as a negative control, and the DNA from the biopsy specimen with *H. pylori* PCR positive result was considered as a positive control. Mastercycler ProS (Eppendorf, Germany) thermocycler was used to amplify the target DNA in the samples. After the electrophoresis of the PCR product in 2% agarose gel at 5 V/cm and ethidium bromide staining, the DNA band was visualized with UV exposure. The sample was determined as *H. pylori-*positive PCR result if DNA band of length 137 bp was seen in gel.

In order to evaluate the quality of DNA extraction, the second human-specific PCR was done to assure the presence of human DNA in each sample. Human *β-*globin gene was the targeted to amplify using the primers KM29 and KM38 following the protocol developed by Saiki et al. [15].

### 2.4. Statistical Analysis

Data were entered in MS Excel 2007 worksheet and further analysed by using SPSS software version 11.5 [16] and R package [17]. Kappa coefficient (*κ*) was used for qualitative analysis of categorical data. McNemar's test was applied to analyse the disagreement between the tests. The chi-squared test was used to analyse the *p* value between the categorical data.

## 3. Results

Out of 52 patients enrolled in this study, majority of the patients were young adults between ages of 20 and 30 years (25%) followed by 60 to 70 years (21.15%) which has been depicted in Table 1. Female patients were, 25 (48.07%), found lesser than male patients, 27 (51.93%). Laboratory analysis of biopsy demonstrated that 9 (17.30%) patients were confirmed *H. pylori* infection as shown in Table 2. Amongst them, 3 (5.77%) cases of *H. pylori* infection were confirmed by microscopy and 8 (15.38%) cases were confirmed by PCR assay.

The spiral- or curved-shaped morphology resembling *H. pylori* was confirmed in 2 (3.84%) Gram-stained biopsies and 1 (1.9%) Giemsa-stained biopsy. We found microscopy positivity in 5.76% patients which could be due to small size of the biopsy. Out of three microscopy-positive cases, the endoscopic examination showed one case of severe erosive duodenitis + antral gastritis and one gastric ulcer + duodenal ulcer. However, one microscopy-positive case had normal mucosa in endoscopic examination. Among microscopy-positive cases, one had consumed PPI in less than two weeks prior to endoscopy with no history of antibiotic intake in any of the patients. The forty-nine microscopy-negative cases have history of PPI consumption in 30 (61.22%) patients and 5 (10.20%) patients had taken antibiotic in less than 2 weeks. The alteration in the morphology of bacteria from the spiral to coccoid form due to consumption of PPI and antibiotics can be responsible for false negativity leading to the possibility of misdiagnosis by the microscopic technique [18, 19].

Among 9 cases of *H. pylori* confirmed by biopsy analysis, endoscopic investigation showed that 32 patients had confirmed gastritis, 6 had duodenitis, and 13 had ulcers as shown in Table 2. But one patient with confirmed *H. pylori* under biopsy analysis had no abnormality in endoscopic observation.

In this study, 44 out of 52 cases had PCR-negative results. In order to rule out the false-negative *H. pylori* PCR results, human *β-*haemoglobin PCR was performed to assure the quality of DNA extraction. Out of three microscopically positive cases, *H. pylori* PCR was also positive in two cases. Among the PCR‐positive cases, endoscopy examination showed two antral gastritis cases, two gastric ulcer cases, one gastric ulcer + duodenal ulcer case, and one severe erosive duodenitis + antral gastritis case, and two gastric ulcer + duodenitis cases. But one case of normal mucosa in endoscopy had also showed PCR-positive test result.

Overall, 9 (17.30%) patients of acid peptic disease (APD) were tested positive by either of the two methods. As yet reported elsewhere, none of the diagnostic assay is stand-alone and universal for disease diagnosis because of several extrinsic and intrinsic limitations [20]. On comparing the different laboratory methods used in detecting *H. pylori*, combination methods using both conventional and molecular techniques have been recommended [21]. Out of 52 patients in the present study, *H. pylori* is confirmed by PCR alone in 6 (11.53%) cases, microscopy alone in 1 (1.92%) case, and combination of PCR and microscopy in 2 (3.84%) cases. Combination of diagnostic assays has proven to be promising in detecting *H. pylori*. In this study, the combination of diagnostic assays microscopy and PCR increased the test positivity from 5.77% (3/52) to 17.31% (9/52).

Two (9.52%) in three microscopy positives and 5 (23.8%) in 8 PCR confirmed *H. pylori* positive had no history of PPI intake. In case of patient with history of antibiotic intake, both diagnostic tests had shown the *H. pylori-*negative results but *H. pylori-*positive test results were demonstrated in patients without antibiotic intake as shown in Table 3. The *H. pylori* microscopy results were 86.54% in agreement with PCR results whereas 13.46% results between both diagnostic tests were in disagreement, but kappa statistical analysis showed that the disagreement was not significant (*p* value = 0.14), as depicted in Table 4. The PCR is superior in diagnosing the presence of the bacteria in gastric biopsy than the microscopy. McNemar's analysis between two assays had shown an agreement of 86.54% (Kappa test, *p*=0.14) which means diagnostic efficiency of both assays was not significantly different.

## 4. Discussion

Several assays have been proposed to detect the *H. pylori* infection; up to date, none of the assay is considered as gold standard for diagnosis of *H. pylori* due to the question in diagnostic precision and feasibility of the available assays [21]. In this study, microscopy and PCR were used as the diagnostic assay, in detecting the presence of the *H. pylori* bacterium in patients with acid peptic disorders using gastric mucosal biopsy. Moreover, bacterial distribution is mostly irregular and/or decreased bacterial load in the available cut specimen. In contrast to our study, Khalifehgholi et al. used Giemsa staining assay and identified *H. pylori* in 77.8% of the specimen [22]. Likewise, Siavoshi et al. found 47.9% *H. pylori* positive by Gram staining [23] and Roy et al. found 65.83% *H. pylori* positive by using modified Giemsa along with hematoxylin + eosin [24]. The results were undoubtedly higher than our findings which could be due to variation in staining techniques and sampling population. Moreover, the study conducted in Nepalese population showed that 67.5% of stomach carcinoma cases were found H. pylori positivity by histopathology and rapid urease analysis [11]. The contrasting results in the aforementioned study could be differences in study population enrolled and the application of diagnostic methods such as histopathological examination using hematoxylin and eosin. However, several reports on advantages of staining by other methods had been reported, and it was not used in the present study [25, 26].

PCR diagnosis by amplifying the conserved gene 23S rRNA has highest performance than other PCR assays [27]. Hence, 23S rRNA PCR was used to identify *H. pylori* infection in this study. In addition, it is efficient to rule out the other neighbouring species which were close to phylogenetic cluster of *Helicobacter* bacteria. The advent of molecular methods for diagnosis of *H. pylori* infection has proven to be a reliable tool as it amplifies the target gene by more than 10^6^ fold, thereby increasing the diagnostic sensitivity and specificity, enabling better clinical management [21]. In addition, it is also capable to detect clarithromycin resistance genotype due to point mutations in the *H. pylori* 23S rRNA gene [28]. Eight (15.38%) out of 52 biopsy specimens detected 23S rRNA which was much lower than that reported by Archampong et al. in Ghana (48.4%) with *cagA* gene [29], Ruparelia et al. in Brazil (50%) with *ureA* + *ureC* gene [30], and Sugimoto et al. (44%) with 16S rRNA [31]. Hundred percent diagnostic accuracy cannot be achieved by the application of single PCR assay [31] since the genomic flexibility between strains of *H. pylori* complicates the choice of target genes [20]. Discrepancy in PCR resulting among the studies could be due to the difference in the type of target genes. In other studies, at least two types of genes were used, either a combination of two virulent genes or a conserved virulent gene. Despite advantages with application of multiple PCR, this study had only single target to amplify *H. pylori* 23S rRNA. Furthermore, other factors such as storage conditions, presence of PCR inhibitor, and repeated thawing and freezing leads to the loss of DNA in the biopsy material [32, 33]. Indeed, low bacterial load, patchy distribution of bacteria in the mucosa, and intake of PPI and antibiotics have been found to negatively influence the outcome of diagnostic tests including PCR. The PCR results were found exactly same as the previous and confirmed the absence of PCR inhibitors. However, none of the patients who had PCR positive took antibiotics for any major or minor sicknesses prior to endoscopy, and 3 (37.5%) PCR-positive cases were taking PPI in less than two weeks. In case of PCR negative, PPI was consumed by 28 (63.63%) patients and antibiotics by 5 patients (11.36%) in less than two weeks. This indicates that the growth of *H. pylori* could be inhibited by uptake of PPI less than two week, but more study with large samples are required to show the significant association.

Shetty et al. showed that diagnostic the sensitivity of microscopy was the highest (54.7%) followed by PCR (54.5%) and rapid urease test (RUT) (48.9%), whereas the culture had sensitivity (29.1%). Among different assays, the PCR had shown the highest sensitivity and specificity [34]. Due to resource constraints, the culture could not be performed and the rate of false positivity in RUT refrained us from performing this test. Moreover, Lim et al. showed that *rpoB* PCR also showed highest positive rate (53.7%) followed by *glmM* PCR (48.8%) [35]. Ruparelia et al. showed that the combination of serology and PCR had the highest sensitivity (100%) rather than RUT (81.81%) in the Indian population [30]. Moreover, recently published report on PCR diagnosis of rhinopharyngeal tumor also had the consistent results with this work [36]. Therefore, in absence of the gold standard assay for identifying *H. pylori*, the combination of diagnostic assays could be applied in order to reduce the false-negative *H. pylori* infection.

## 5. Conclusion

Although PCR is more sensitive assay to detect *H. pylori* infection than microscopy, it is not yet considered as the gold standard assay. Therefore, in order to improve the diagnostic accuracy, we recommend the combination of microscopy and PCR assay for effective monitoring of *H. pylori* infection in endemic sites.

## Figures and Tables

**Table 1:** Demographic and laboratory analysis (*n* = 52).

Age (years)	Frequency
20–30	13 (25%)
30–40	8 (15.38%)
40–50	8 (15.38%)
50–60	7 (13.46%)
60–70	11 (21.15%)
70–80	5 (9.63%)
*Gender*	
Female	25 (48.07%)
Male	27 (51.93%)
*Laboratory test*	
Microscopy positive	3 (5.77%)
PCR positive	8 (15.38%)

**Table 2:** Comparison of endoscopy findings and positive *H. pylori* tests (*n* = 52).

Endoscopy finding	No. of cases (%)	*H. pylori-*positive case (%)
Normal	1 (1.92)	1 (11.11)
Gastritis	32 (61.54)	2 (22.22)
Duodenitis	6 (11.54)	1 (11.11)
Ulcers	13 (25.0)	5 (55.56)
Total	52	9

**Table 3:** History of PPI and antibiotic intake in comparison with diagnostic test positive for *H. pylori*.

History of PPI intake	Microscopy positive	PCR positive
Yes (*n* = 31)	1 (3.22%)	3 (9.67%)
No (*n* = 21)	2 (9.52%)	5 (23.8%)
Total (*n* = 52)	3 (5.77%)	8 (15.3%)
*p* value	0.02^*∗*^	0.32
*History of antibiotic intake*		
Yes (*n* = 5)	—	—
No (*n* = 47)	3 (6.38%)	8 (17.0%)
Total (*n* = 52)	3 (5.76%)	8 (15.3%)
*p* value	1	0.73

^*∗*^Statistical significant at 0.05.

**Table 4:** Comparison of diagnostic results for *H. pylori* (*n* = 52).

Microscopy	PCR	Frequency	Agreement^*∗*^	Disagreement^$^	Kappa test (*p* value)
Positive	Positive	2	86.54%	13.46%	0.14
Positive	Negative	1			
Negative	Positive	6			
Negative	Negative	43			

^*∗*^Agreement between microscopy and PCR. ^$^Disagreement between microscopy and PCR.

## Data Availability

This is a hospital-based study. Samples were collected during the routine diagnostic procedure, and the samples were used to evaluate the performance of PCR to find their role in the implementation of diagnostics in hospital. Therefore, the data of the analysis are available upon the request from the corresponding author or head, department of Microbiology (hod.microbiology@bpkihs.edu), BP Koirala Institute of Health Sciences, Dharan, Nepal.
